# Structural and Electronic Transitions in Liquid FeO Under High Pressure

**DOI:** 10.1029/2022JB025117

**Published:** 2022-11-05

**Authors:** Guillaume Morard, Daniele Antonangeli, Johann Bouchet, Attilio Rivoldini, Silvia Boccato, Francesca Miozzi, Eglantine Boulard, Hélène Bureau, Mohamed Mezouar, Clemens Prescher, Stella Chariton, Eran Greenberg

**Affiliations:** ^1^ CNRS IRD IFSTTAR ISTerre Université Grenoble Alpes Université Savoie Mont Blanc Grenoble France; ^2^ Muséum National d'Histoire Naturelle UMR CNRS 7590 Institut de Minéralogie de Physique des Matériaux et de Cosmochimie IMPMC Sorbonne Université Paris France; ^3^ CEA DAM DIF Bruyères Le Chatel France; ^4^ CEA DES IRESNE DEC Cadarache Saint‐Paul‐Lez‐Durance France; ^5^ Royal Observatory of Belgium Brussels Belgium; ^6^ Now at Earth and Planets Laboratory Carnegie Institution for Science DC Washington USA; ^7^ ESRF Grenoble France; ^8^ DESY Hamburg Germany; ^9^ Institute of Earth and Environmental Science University of Freiburg Freiburg Germany; ^10^ GSECARS The University of Chicago IL Chicago USA; ^11^ Now at Applied Physics Division Soreq NRC Yavne Israel

**Keywords:** high pressure, liquid FeO, thermal equation of state, liquid structure

## Abstract

FeO represents an important end‐member for planetary interiors mineralogy. However, its properties in the liquid state under high pressure are poorly constrained. Here, in situ high‐pressure and high‐temperature X‐ray diffraction experiments, ab initio simulations, and thermodynamic calculations are combined to study the local structure and density evolution of liquid FeO under extreme conditions. Our results highlight a strong shortening of the Fe‐Fe distance, particularly pronounced between ambient pressure and ∼40 GPa, possibly related with the insulator to metal transition occurring in solid FeO over a similar pressure range. Liquid density is smoothly evolving between 60 and 150 GPa from values calculated for magnetic liquid to those calculated for non‐magnetic liquid, compatibly with a continuous spin crossover in liquid FeO. The present findings support the potential decorrelation between insulator/metal transition and the high‐spin to low‐spin continuous transition, and relate the changes in the microscopic structure with macroscopic properties, such as the closure of the Fe‐FeO miscibility gap. Finally, these results are used to construct a parameterized thermal equation of state for liquid FeO providing densities up to pressure and temperature conditions expected at the Earth's core‐mantle boundary.

## Key Points

- Shortening of the Fe‐Fe bond length in liquid FeO is correlated with the insulator to metal transition in solid state
- Liquid FeO density smoothly evolves from high spin to low spin between 60 and over 150 GPa
- Changes in the microscopic structure are related to macroscopic properties, such as the closure of the Fe‐FeO miscibility gap

## 1 Introduction

Investigation of the structural and electronic properties of solid and liquid FeO at high pressures and temperatures is of great interest in geophysics and planetary sciences, as well as in condensed‐matter physics. FeO is a typical Mott insulator under ambient conditions and a prototypical highly correlated transition metal oxide. Different electronic and structural transitions occur in FeO under compression at ambient temperature. Along with other transition metal oxides, it undergoes a spin crossover and it metalizes under pressure (Ohta et al., 2012). One of the open questions is to understand if the metalization and the transition from high spin to low spin state in the solid state are simply concomitant or more intricately related (Greenberg et al., 2020; Leonov, 2015; Ozawa, Hirose, et al., 2011). Similar to the case of other crystalline planetary materials, the spin crossover has received much attention in the solid state (see reviews by Badro [2014] and Lin et al. [2013]) but its effects in the liquid phase are still relatively poorly known (Holmstrom & Stixrude, 2016). An improved knowledge of physical properties of iron alloys in their liquid state under extreme conditions is required to understand the composition and properties of planetary cores, as well as to constrain scenarios of planetary differentiation.

FeO represents an important end‐member, as O is expected to be a major light element in Earth's core (Badro et al., 2014; Hirose et al., 2013; Poirier, 1994), but also in Mars' core (Stähler et al., 2021; Tsuno et al., 2011; Yoshizaki & McDonough, 2020). The binary Fe‐FeO system evolves from a large immiscible system at ambient pressure to a binary eutectic system above 40 GPa (Morard, Andrault, et al., 2017; Morard, Nakajima, et al., 2017; Oka et al., 2019; Ringwood & Hibberson, 1990; Tsuno, Terasaki, et al., 2007). The reduction of the miscibility gap with increasing pressure is expected to significantly affect core differentiation mechanisms, depending on planetary size. For example, the amount of siderophile elements in the Earth's mantle indicates a pressure exceeding 50 GPa at the bottom of the primitive magma ocean (Fischer et al., 2015; Siebert et al., 2013), and thus a high oxygen solubility in the metallic phase. Conversely, an early magma ocean in Mars would have reached a maximal pressure of ∼14 GPa and temperature of ∼2100 K (Rai & Van Westrenen, 2014; Righter & Chabot, 2011), within the liquid Fe‐FeO miscibility gap. Under this pressure and temperature conditions, the solubility of oxygen in pure iron is relatively low, with a maximum of 4 wt%O (Tsuno, Ohtani, & Terasaki, 2007). Thus, beside the nature of the accreting material and the overall redox conditions, difference in planetary size plays a key role in controlling the oxygen partitioning during the planetary differentiation.

Iron oxides are also important to understand the mineralogy of planetary mantles. Ferropericlase (Mg,Fe)O is the second most abundant mineral in the Earth's lower mantle and an FeO‐enriched layer, formed either at the end of magma ocean crystallization (Boukaré et al., 2015) or later on from mantle‐core interactions (Hirose et al., 2017; Trønnes et al., 2019), is possibly still present at the core‐mantle boundary. It is intriguing and noteworthy to note that FeO as end‐member is as relevant for planetary mantles as it is for planetary cores, and that exchange of FeO between mantle and core can be one of the main processes occurring during core‐mantle interaction.

The electronic properties of FeO have been a long‐standing study case in physics with potential implications in geophysics. At ambient pressure, FeO is an insulator with anti‐ferromagnetic spin ordering. In the B1 structure, an isostructural transition from insulator to metal occurs between 30 and 80 GPa at high temperature. This transition has been observed through changes in emissivity (Fe_0.94_O) (Fischer, Campbell, Shofner, et al., 2011) and electrical conductivity properties (Fe_0.96_O) (Ohta et al., 2012). The relation between modifications in the electrical conductivity and structure and/or magnetic properties is still unclear. Changes in the spin state of Fe atoms, related with the magnetic properties, have been postulated to be at the origin of the drastic volume reduction and transition from r‐B1 structure to B8 structure under cold compression (Ozawa, Hirose, et al., 2011). Only recently, the complex interplay between structure, spin state, conductivity and elastic properties in solid FeO at high temperature has started to be constrained (Greenberg et al., 2020) but this is far less understood in the liquid phase.

In the present study, we investigated the local atomic structure of liquid FeO from 13 GPa–2600 K to 85 GPa −3800 K by in situ X‐ray diffraction (XRD) using Laser‐Heated Diamond Anvil Cell (LH‐DAC). By comparing our experimental results with ab initio molecular dynamics (AIMD) simulations, we were able to identify the physical mechanism responsible for the closure of the Fe‐FeO miscibility gap in the progressive approaching of the Fe‐Fe bond length in liquid Fe and liquid FeO, with the two converging around 40 GPa. This change takes place at the same pressure at which the insulator‐metal transition occurs in the solid state. In addition, densities determined with the thermal equation of state (EoS) calculated for liquid FeO from thermodynamic modeling are observed to smoothly distance from the densities calculated for the magnetic liquid at pressures above 60 GPa and to approach densities calculated for the non‐magnetic liquid above 150 GPa, indicating a mixed spin state over this pressure range.

## 2 Methods

### 2.1 High Pressure Experiments

Two different types of starting materials were used in the present study: (a) Fe_x_O (*x* = 0.92 following McCammon and Liu [1984]) synthesized under reducing conditions from hematite at 1200°C for 24 hr (courtesy of Prof S. Jacobsen from Northwestern University) was used during the experiment at the 13‐IDD beamline at APS, Argonne, USA; (b) commercial Fe_x_O powder from Alfa Aesar measured at *x* = 0.937 following (McCammon & Liu, 1984), very close to the sample used in (Fischer, Campbell, Shofner, et al., 2011) (*x* = 0.94) was used during experiments on the ID27 beamline, at ESRF, Grenoble, France. Although the obtained results seem to be independent of the starting material, for the sake of completeness, in Table 1 we specify the used samples in each experimental run. In particular, melting temperatures, liquid structure and solid Fe_x_O compressibility of Fe_0.92_O and Fe_0.94_O are within mutual uncertainties.

**Table 1:** Structural Analysis for Liquid FeO Measured by In Situ X‐Ray Diffraction

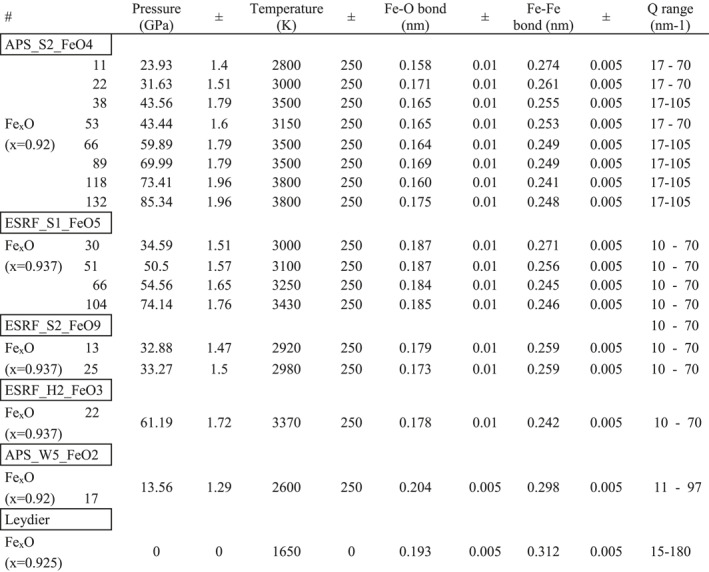

*Note.* Pressure and temperature are indicated for each run. Position of Fe‐O and Fe‐Fe bonds are measured on total *g*(*r*) by Gaussian fit of the first and second oscillations (using Fityk software [Wojdyr, 2010]).

The experiments were conducted using membrane driven Le Toullec type DAC, with 250 μm flat or 150/300 beveled diamonds to generate high pressures. Samples were crushed into smaller pieces and powders or flakes, with an initial thickness of ∼10 μm, were placed in the experimental chamber drilled in a Re gasket, between two pellets of KCl, acting as pressure medium, thermal insulator, and pressure marker following the method developed in Campbell et al. (2009).

Angle dispersive X‐ray diffraction (XRD) experiments using double‐sided laser heating diamond anvil cells were performed on two different beamlines: ID27 beamline (ESRF, Grenoble, France) and 13ID‐D (APS, Argonne, USA).

The experimental set‐up on ID27 beamline (Mezouar et al., 2005) consists of two continuous Nd:YAG fiber lasers (TEM 00) providing a maximum total power of 200 W. Diffraction signal was collected using a MAR CCD detector for an incoming energy of 33 keV. Laser spot size was adjusted around 20 μm diameter, while X‐ray focusing was fixed to 3 × 3 μm^2^. Temperature was measured on both sides before and after XRD acquisition, via spectroradiometric method, using reflective collecting optics (Giampaoli et al., 2018; Mezouar et al., 2017). During heating runs, temperature was monitored only from the upstream side and kept stable by tuning laser power when required (exposure time was kept at 5 s in the solid phase, while for liquid XRD, exposure time was increased to 30–60 s).

The experimental set‐up on 13 ID‐D beamline consists of a double‐sided LH‐DAC combined with a multichannel collimator (MCC) set‐up (Mezouar et al., 2002; Morard et al., 2011; Weck et al., 2013) and a DECTRIS Pilatus 3XM CdTe detector (version 300K‐W), using an energy of 37.077 keV for the incoming X‐ray beam. Temperature was measured in situ from both sides using refractive optics with the experimental set up detailed in Shen et al. (2006). Using the MCC combined with LH‐DAC reduces the incoherent signal coming from the diamonds and allows to increase the signal‐to‐noise ratio (Prescher et al., 2017). Minimum exposure time required by data collection with MCC slits was 60 s.

The error bar on the temperature for the melting point determination is estimated at ±150 K, accounting for radial and axial *T* gradients, as in Morard, Andrault, et al. (2017) and Morard, Nakajima, et al. (2017). Temperature uncertainties for liquid XRD patters are ±250 K to also account for *T* fluctuations during the longer exposure time. Pressure was calibrated using the thermal EoS of the KCl pressure medium (Dewaele et al., 2012) and uncertainties are estimated from the width of the KCl diffraction peaks (±1 GPa) and the uncertainty in the temperature measurement, following the method developed in Campbell et al. (2009). The small thermal expansion of KCl makes it an excellent pressure marker under high temperature.

The fit of the melting curve was performed combining multiple data sets (first liquid and last solid by in situ XRD obtained in this study, first liquid by change of the optical properties [Fischer & Campbell, 2010]) within a Monte Carlo approach: 50 replications of 500 independent sets of *P*‐*T* points were used, where the *P*‐*T* points are distributed around the experimental *P*‐*T* values with a normal distribution with standard deviation of 10% for both *P* and *T*.

Diffuse scattering from the liquid was analyzed following previously detailed methods (Eggert et al., 2002; Morard et al., 2013), with specific interest in the liquid structure, not in the density determination. In order to minimize the effect from limited range of the wave vector *Q*, a variable Lorch modification function is used, following the parametrization presented in Skinner et al. (2013). Obtaining a high quality XRD signal for a low scattering liquid sample such as FeO is extremely difficult. Depending on the experimental set‐up (availability of MCC setup or not), but also from pattern to pattern, the quality of the signal, in particular at high angle, could vary. Therefore, different *Q* ranges were here considered (indicated for each pattern). The chosen *Q*‐range affects mainly the width of oscillations in real space, without a strong effect on their position. A more detailed discussion on *Q* range effect can be found in Morard, Andrault, et al. (2017) and Morard, Nakajima, et al. (2017). The liquid structure results show no obvious correlation with the used *Q* range.

Analysis of the XRD patterns of the quench of the FeO liquid indicates the absence of evident carbon contamination as the main diffraction peaks can be indexed by FeO B1 phase and KCl pressure transmitting medium, without visible contribution by carbide phases.

### 2.2 Numerical Simulations

Ab initio calculations were performed using the ABINIT package (Gonze et al., 2020). We used a cubic simulation cell of 64 atoms to simulate the B1 structure (8 units) and the liquid, similar to the one used in a recent work on ferropericlase (Holmström & Stixrude, 2015, 2016). In coherence with previous simulations (Dewaele et al., 2006; Morard et al., 2018), PAW atomic data with a short cutoff radius (2.0 a.u.) and including 16 electrons in the valence were used. We employed a plane wave energy cutoff equal to 544 eV to span the wavefunctions, and a 2 × 2 × 2 **k** points mesh to sample the irreducible Brillouin zone.

It is well known that in FeO the electronic correlations between the 3*d* electrons are strong and cannot be described using the local density approximation (LDA) or the generalized gradient approximation (GGA). In disagreement with experiments, GGA calculations (Leonov, 2015) give a metallic ground state with an equilibrium lattice constant of 7.74 a.u, about 6%–7% smaller than the measured one. To capture the correlations effects we must go beyond this treatment of the electronic density and use extensions such as +*U* (Holmström & Stixrude, 2015) or dynamical mean field theory (DMFT) (Leonov, 2015; Ohta et al., 2012; Zhang et al., 2017). GGA + DMFT calculations reveal a high‐spin to low‐spin transition within the B1 structure upon compression accompanied by an insulator to metal transition around 70 GPa at 0 K. The crossover is very broad (Leonov, 2015; Zhang et al., 2017) with a width increasing with temperature (Zhang et al., 2017). Unfortunately, the DMFT method is computationally too expensive to be used with supercells and ab initio molecular dynamics (AIMD), so here we used the GGA + *U* method where the Coulomb interaction is treated in static mean‐field theory. This method cannot capture true many body effects but is tractable with AIMD. It reproduces the band gap of the ground state (Gramsch et al., 2003; Mazin & Anisimov, 1997) and we obtain a value of 8.18 a.u for the equilibrium lattice constant of the B1 structure, close to the value of 8.36 a.u obtained with GGA + DMFT (Leonov, 2015). A similar approach was also applied on crystalline and liquid (Mg,Fe)O where a spin crossover takes place with pressure as in FeO. For *U* we used two values 5 and 7 eV, in the range of the values used in previous calculations (Leonov, 2015; Zhang et al., 2017). It was shown recently using self‐consistent LDA + *U*_*sc*_ method that this parameter can strongly depend on pressure, structure and spin state (Sun et al., 2020). The temperature effect on the *U* parameter was not considered in this work.

Concerning the magnetism of the Fe atoms, we performed two types of calculations: non‐spin (no magnetism) and spin polarized. In the second case, at the beginning of the calculations, the Fe atoms were in a high spin state. We also tried several configuration where some of the Fe atoms were randomly in high or low spin state to simulate the spin crossover (Holmstrom & Stixrude, 2016). During the simulations, the spin moments were free to evolve and not fixed at their starting values.

Finally, we also performed calculations with a supercell of 128 atoms to study the size effect on pressure. The difference with the smaller cell (64 atoms) were lower than one GPa for a similar fixed volume.

### 2.3 Thermodynamic Calculations

To derive an equation of state of liquid FeO, we use the equation of state of solid B1 FeO and the melting curve deduced in this study.

Along the melting temperature $T_{m}$ the Gibbs energies of B1 ($G_{FeO}^{B1})$ and liquid ($G_{FeO}^{l})$ FeO are equal:

(1) $$G_{FeO}^{B1}\left(p,T_{m}\right)=G_{FeO}^{l}\left(p,T_{m}\right)$$

The Gibbs energy of both phases is related to the Helmholtz energy by the following Legendre transformation (Poirier, 2000):

(2) G(p,T)=F(V,T)+p(V,T)V

The Helmholtz free energy can be written as

(3) $$F\left(V,T\right)=U_{0}+E_{0}\left(V\right)+F_{th}\left(V,T\right)-F_{th}\left(V,T_{0}\right)+F_{el}\left(V,T\right)-F_{el}\left(V,T_{0}\right)-a_{s}R\left(T-T_{0}\right)$$

where $U_{0}$ is the reference energy at reference temperature $T_{0}$ and volume $V_{0}$, $E_{0}\left(V\right)$ is the contribution from compression at $T_{0}$, $F_{th}$ represents the thermal part of the free energy, $F_{el}$ the electronic part, and the part proportional to $a_{s}$ accounts for residual entropy at 0 K in the liquid phase.

The EoS of B1 FeO is based on the Mie‐Grüneisen‐Debye formulation using a third order Birch‐Murnaghan equation for the cold compression part (Ita & Stixrude, 1992), without explicitly considering the electronic contribution. The EoS of solid B1‐FeO is discussed in Section 3.

For the EoS of liquid FeO, we use the formulation for l‐Fe used in Dorogokupets et al. (2017). All the parameters for the liquid FeO have been estimated by fitting the expression of the Helmholtz energy to $G_{FeO}^{B1}\left(p,T_{m}\right)$, the heat capacity at constant pressure $C_{p}\left(T_{0}\right)=68.15\frac{J}{Kmol}$ (NIST), and measured volumes at 1 bar (Xin et al., 2019). The fitting parameters are presented in Table S2 of Supporting Information S2.

## 3 Results

### 3.1 No Signature of the Insulator‐Metal Transition on Solid B1‐FeO

**Figure 1:**
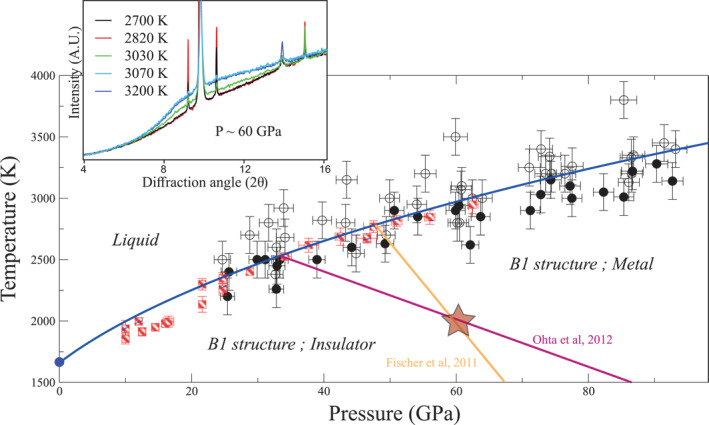
Melting curve of FeO. Full circle symbol indicates the last solid, empty circle symbol the first liquid observed upon heating runs (data presented in Table S1 of Supporting Information S2). Previous measurements of the melting temperature of FeO, based on the changes of optical properties upon heating, are indicated by red hashed square symbols (Fischer & Campbell, 2010). Fit of the melting curve to a Simon‐Glatzel's equation (Simon & Glatzel, 1929) yields the following parameters (*a* = 14.89 ± 4.73; *c* = 2.77 ± 0.51; *P*_0_ = 0 GPa; *T*_0_ = 1650 K). Proposed phase boundaries of the isostructural transition between metallic and insulator B1 structure occurring in the solid state (Fischer, Campbell, Lord, et al., 2011; Ohta et al., 2012) are also shown. The star symbol represents the intersection between the 2000 K isotherm and the metal to insulator transition, occurring around 62 GPa, according to both Fischer, Campbell, Shofner, et al. (2011) and Ohta et al. (2012). Melting temperature of FeO at 1 bar is indicated by a round symbol. Inset: Example of appearance of diffuse scattering with increasing temperature, used as melting criteria.

The melting curve of B1 FeO structure has been determined by using the first appearance of diffuse signal from liquid FeO as melting criteria (Figure 1, inset). As shown in Figure 1, the melting curve, here extended up to pressures above 90 GPa, is in excellent agreement with previous lower pressure measurements performed using a different melting criteria (emissivity vs. temperature) (Fischer & Campbell, 2010). When considering our data set together with results from previous studies (Fischer, Campbell, Lord, et al., 2011), we do not observe a clear discontinuity in the melting curve in the 30–60 GPa range. The absence of discontinuity could be possibly associated to the gradual insulator to metal transition in liquid FeO (Figure 1). We note however that the global uncertainties on the melting diagnostics and on the related pressure and temperature metrology do not currently allow determining slope changes resulting in a temperature difference smaller than ∼200 K.

**Figure 2:**
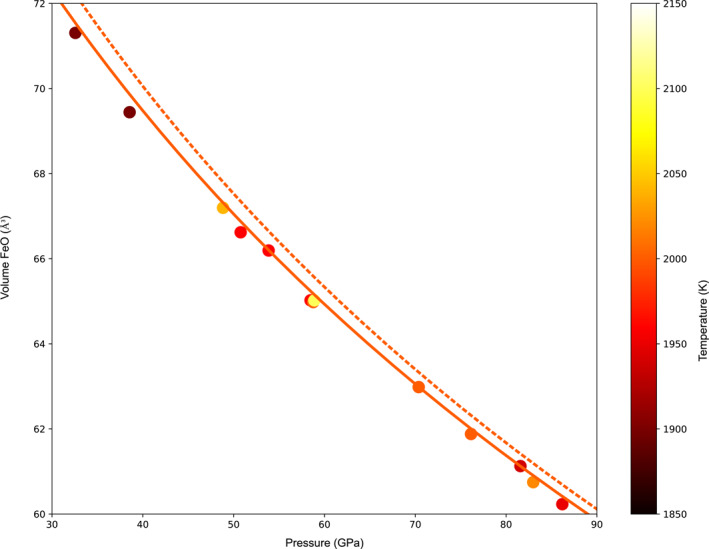
Experimental volumes of solid FeO in the B1 structure along a 2000 K isotherm. The different volumes are taken from this experiment and from Fischer, Campbell, Shofner, et al. (2011). The circles correspond to volumes between 1850 and 2150 K (the whole data set of solid volumes in the B1 structure is given in Table S3 of Supporting Information S2, as well as the associated error bars on temperature, pressure and volume). Full line corresponds to thermal equation of state (EoS) parametrized in the present work, whereas dotted line corresponds to the thermal EoS proposed by Fischer, Campbell, Shofner, et al. (2011). A single equation of state well describes the entire data set, and no clear discontinuity is visible around the metallic transition, occurring around 60 GPa at this temperature (see also Figure 1).

In order to investigate the effect of the insulator‐metal transition in the solid state, we measured the volumes of FeO in the B1 structure as a function of pressure and temperature (Table S3 in Supporting Information S2). A thermal equation of state, based on the third order Birch Murnaghan equation and Mie Grüneisen‐Debye formalisms, was determined following the parametrization used by Fischer, Campbell, Lord, et al. (2011). In this previous study, volumes were measured in a complementary pressure range (∼90–160 GPa in Fischer, Campbell, Lord, et al., 2011 and ∼30–90 GPa here). For isothermal compression at ambient temperature, we assumed the parameters used in Fischer et al. (*V*_0_ = 81.41 Å^3^, *K*_0_ = 149.4 GPa; *K*_0_′ = 3.6) and the Debye temperature Ɵ_0_ was set to 417 K (Stixrude & Lithgow‐Bertelloni, 2007). Other parameters entering in the thermal model were fitted combining the present data set and previous measurements (Fischer, Campbell, Shofner, et al., 2011), leading to a slightly different set of values (*γ*_0_ = 1.05; *q* = 0.123) (Figure S1 in Supporting Information S1). Results of the obtained fit along an isotherm at 2000 K are illustrated in Figure 2 together with measured volumes in the 1900–2100 K range.

A single equation of state well reproduces the experimental volumes over the whole pressure range, with no discontinuity at pressures corresponding to the insulator to metal transition. Thus, from our results, we could neither evidence a change in volume in the solid phase (Figure 2) nor detect a change in the slope of the melting curve (Figure 1) in the pressure range where the transition from insulator to metal in the B1 structure occurs.

### 3.2 Structural Evolution of Liquid FeO Under High Pressure

**Figure 3:**
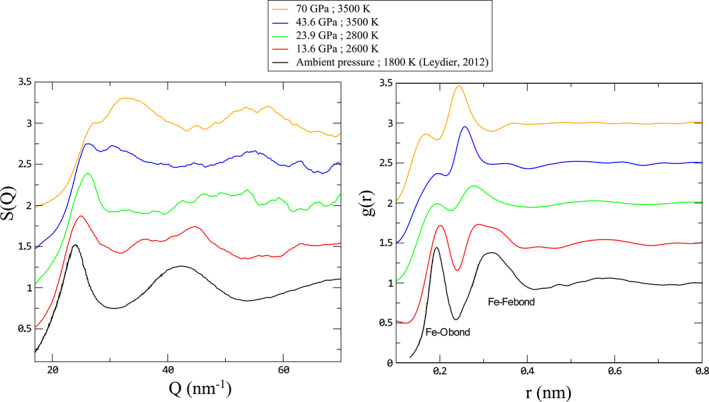
Structure factor *S*(*Q*) and radial distribution function *g*(*r*) of liquid FeO at high pressure. Evolution of the structure factor *S*(*Q*) with increasing pressure highlights the progressive appearance of a shoulder around 35 nm^−1^ that becomes a prominent feature above 40 GPa. The corresponding *g*(*r*) show the progressive shift toward lower distance of the second oscillation, assigned mainly to the Fe‐Fe bond.

Evolutions of structure factors *S*(*Q*) (*Q* is the wave vector in nm^−1^) and radial distribution functions *g*(*r*) (*r* is the distance from a reference atom in nm) obtained for FeO liquid between ambient pressure (Leydier, 2010) and 70 GPa are presented in Figure 3 (subset of data obtained over a larger pressure range up to 85 GPa and 3800 K). The relative temperature difference to the melting line is almost constant for the XRD patterns of liquids recorded here (between 200 and 500 K above the melting curve). Therefore, the effect of temperature on the liquid structure may be relatively constant over the investigated pressure range. The structural evolution presented here, that is, the shift toward higher *Q* of the main peak and the appearance of a new contribution around 35 nm^−1^ (Figure 3), are driven by the pressure increase.

**Figure 4:**
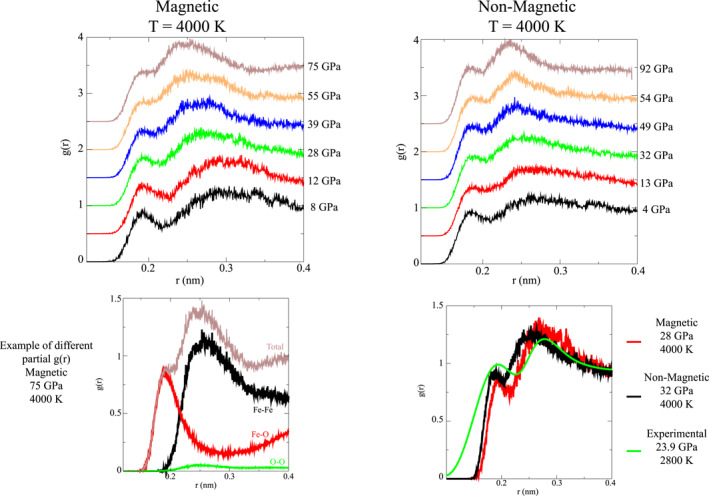
Calculated radial distribution function *g*(*r*) for liquid FeO at high pressure. Top: Total *g*(*r*) calculated at 4000 K for increasing pressures, using magnetic (left) and non‐magnetic (right) potentials. Bottom left: Total and partial *g*(*r*), illustrating the distinct contributions of Fe‐Fe and Fe‐O bonds. The different partial *g*(*r*) are weighted for their atomic number following (Waseda, 1980). *P*‐*T* conditions and bonds length are presented in Table 1. Bottom right: Comparison between experimental and theoretical *g*(*r*).

To provide a better interpretation of the experimentally determined radial distribution function *g*(*r*), *ab initio* molecular dynamics (AIMD) calculations were performed at similar *P*‐*T* conditions (Figure 4). For the “non‐magnetic” results, non‐spin polarized simulations were performed, with a magnetic moment of the Fe atoms forced to 0, simulating the absence of spin polarization of the electrons. For “magnetic” results, the Fe atoms were considered in a high spin state, as given by Hund's first rule and expected for the insulator behavior at lower pressure (Leonov, 2015).

The contributions of the Fe‐Fe and Fe‐O bonds can be clearly identified in the computed radial distribution functions *g*(*r*) (Figure 4), whereas it is difficult to identify accurately the position of the Fe‐O contribution in the experimental data set, potentially due to the short *Q* range of the measurements. In the AIMD calculations, the Fe‐O bond distance is almost constant with pressure, decreasing only by ∼2% between 4 and 92 GPa (Tables S4 and S5 in Supporting Information S2). Conversely, the Fe‐Fe bond distance strongly decreases, as visible both on the experimental and theoretical *g*(*r*) (Figures 3 and 4). The contribution of O‐O bond to the total *g*(*r*) is minor and cannot be resolved, as the weight is proportional to the atomic number. For completeness, the partial O‐O *g*(*r*)s obtained by calculations are presented in Figure S3 of Supporting Information S1. Differently from calculations performed on liquid Fe_2_O_3_ under high temperature (Misawa et al., 2014) our results show no indication of O‐O dimer formation.

Guided by results from AIMD calculations, experimental *g*(*r*) were further analyzed in order to quantitatively assess the structural evolution of liquid FeO with pressure. Peak positions of the different *g*(*r*) contributions were refined by performing a Gaussian fit using Fytik software (Wojdyr, 2010) (Table 1; Tables S4 and S5 in Supporting Information S2), with the two first Gaussians assigned to Fe‐O and Fe‐Fe bonds respectively, and a sigmoid used to fit the rest of the *g*(*r*) (Example of fit is shown in Figure S2 of Supporting Information S1). In order to better compare with the experimental *g*(*r*), a similar analysis was also performed for the total *g*(*r*) obtained by ab initio calculations.

Fe‐Fe bond length evolution with pressure from experimental and ab initio results are presented in Figure 4. In the 0–100 GPa pressure range, we observe a strong decrease of the Fe‐Fe distance, from 0.313 nm at ambient pressure to ∼0.24 nm at ∼90 GPa for experimental measurements, a variation larger than 20%. This evolution is not monotonic, with a first steep decrease between 0 and 40 GPa, followed by a smaller decrease.

Experimental measurements follow trend similar to that of magnetic AIMD calculations up to ∼40 GPa, with a strong decrease by more than 15% for the experimental data set. In contrast, Fe‐Fe bond length in pure liquid Fe, deduced from recent high pressure experiments (Kuwayama et al., 2020), follows the pressure evolution of the non‐magnetized simulations of our study, as expected for the metallic state of liquid Fe.

**Figure 5:**
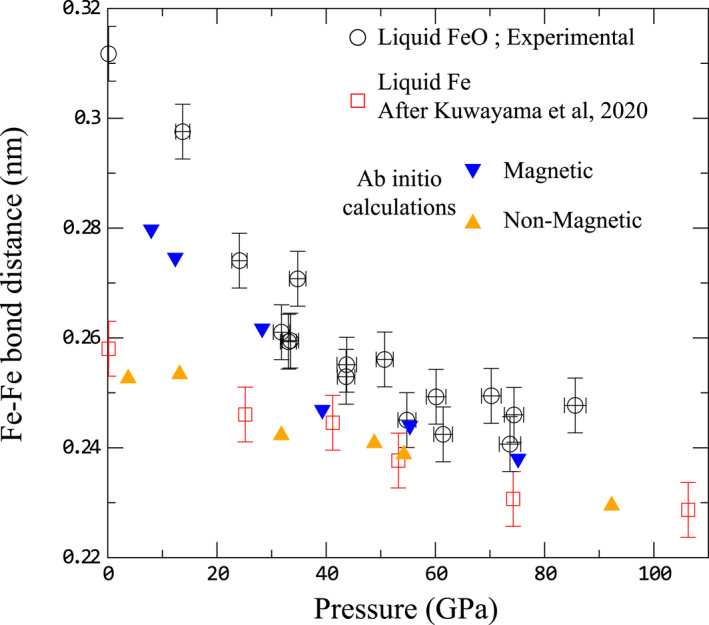
Fe‐Fe bond distance in liquid FeO and pure liquid Fe as a function of pressure. Circles are experimental results (Table 1), blue downward triangles and orange upward triangles are results of fits to the ab initio calculations, magnetic (Table S4 in Supporting Information S2) and non‐magnetic (Table S3 in Supporting Information S2) simulations, respectively. Red squares are Fe‐Fe bond in pure liquid Fe at high pressure (Kuwayama et al., 2020), and at ambient pressure (Waseda & Suzuki, 1970).

Above 40 GPa, the Fe‐Fe bond length in FeO starts to be less affected by further pressure increase, and the structural difference between magnetic and non‐magnetic liquids is smaller. Current uncertainties, largely due to the short Q‐range of our experimental data, do not allow to unequivocally infer a complete transition toward the non‐magnetic structure. However, the Fe‐Fe bond length in liquid Fe and liquid FeO get very close above 40 GPa (Figure 5). It should be noticed that this pressure corresponds to the intersection of the insulator –metal transition intersection with the melting line (Figure 1).

This structural evolution of liquid metals coincides with experimental observations of the closure of the miscibility gap in the Fe‐FeO binary system (between 23 and 38 GPa) (Oka et al., 2019). Close values of bond distances favors solubility (Hume‐Rothery rules [Hume‐Rothery & Powell, 1935]), thus our observations provide the direct microscopic explanation for such a significant change in the phase diagram. Closure of the miscibility gap related with changes in the atomic arrangement of the liquids has been already evidenced in the ternary Fe‐S‐Si ternary system (Morard et al., 2007, 2008). These studies evidenced how the structural evolution from a disordered toward a compact structure for liquid Fe‐S alloys around 15 GPa (Morard et al., 2007) makes Fe‐S alloys above this pressure structurally compatible with liquid Fe‐Si alloys of similarly compact structure (Morard et al., 2008), and this can be directly correlated with the closure of the Fe‐S‐Si miscibility gap under similar pressure (Sanloup & Fei, 2004).

### 3.3 Thermodynamic Modeling of Liquid FeO Thermal Equation of State

**Figure 6:**
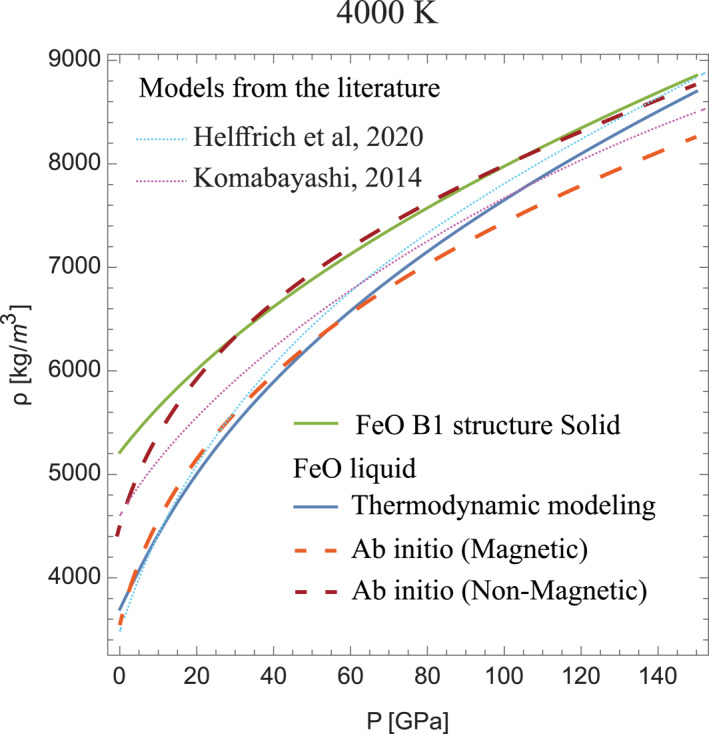
Density of solid and liquid FeO along an isotherm at 4000 K. The green solid line represents the isotherm for solid FeO in the B1 structure according the thermal EoS established in this study and the blue solid line the isotherm for liquid FeO according to thermodynamic modeling (see Section 2). The electronic contribution in our model (Dorogokupets et al., 2017) is fixed so to reproduce the density at ambient pressure (Xin et al., 2019). The two dashed lines represent the density of magnetic (light red, lower density) and non‐magnetic (dark red, higher density) liquid FeO obtained interpolating ab initio data set (Tables S4 and S5 in Supporting Information S2). Two models from the literature (dotted lines) are also represented (Helffrich et al., 2020; Komabayashi, 2014).

Using the hereby experimentally determined thermal EoS for solid FeO in the B1 structure and the FeO melting curve (Figure 1), we were able to calculate the EoS of liquid FeO based on thermodynamic modeling (see Section 2) (Figure 6). Unlike for solid FeO, to describe liquid FeO, we use an expression for the Helmholtz energy including a free electron contribution (e.g., Dorogokupets et al., 2017). This contribution is essential in order to reproduce the low pressure and high temperature behavior of the density shown by our magnetic AIMD calculations and to yield density in agreement with the 1 bar experimental results of Xin et al. (2019) or the density of liquid FeO deduced by Helffrich et al. (2020) at 1 bar and 3000 K (Figure 6).

Comparison of thermodynamic and AIMD calculations (with different spin‐polarization properties) allows us to estimate how the density evolution with pressure relates to spin transition in the liquid. Below 60 GPa, spin polarization in liquid FeO is required in the calculation to reproduce the experimentally observed Fe‐Fe bond length (Figure 5), effectively lowering the density, in agreement with thermodynamic modeling and expected density at ambient pressure (Xin et al., 2019) (Figure 6). Above 140 GPa, the extrapolated density of liquid FeO determined from the thermodynamic calculations tends toward the non‐magnetic density values (Figure 6). At intermediate pressures, the density of liquid FeO according to the thermodynamic modeling smoothly evolves between the two magnetic states. A more quantitative comparison and eventual modifications in the thermodynamic parameters to accurately describe the transition regime would require experimental validation of the density provided by calculations and data more sensitive to the magnetic transition than the density or the melting curve.

The difference between the present liquid FeO thermal EoS and the one proposed in Komabayashi (2014) (Figure 6) is mainly related to the different melting curve for FeO used to calculate the liquid EoS. In this previous study (Komabayashi, 2014), Fe and FeO melting curves were assumed to cross, with FeO melting temperature higher than those of pure Fe above ∼20 GPa. Our experiments show that the FeO melting curve is lower than the Fe melting curve over the entire pressure range here investigated, thus requiring an updated parametrization for the liquid FeO thermal equation of state. The difference between our model and the recent one by Helffrich et al. (2020) is relatively small (Figure 6) as this second work calculated a FeO melting curve closer to Fischer and Campbell (2010), and thus in agreement with the present study.

## 4 Discussion

### 4.1 Insulator/Metal Transition and Spin Transition in FeO

Spin transition has been reported to affect the elastic properties of solid ferropericlase, (review in Badro [2014]) with smaller or larger effects depending on considered frequencies and used techniques (Antonangeli et al., 2011; Crowhurst et al., 2008; Marquardt et al., 2018). The spin crossover is also expected in the liquid state, although with a negative Clapeyron slope (Holmstrom & Stixrude, 2016), opposite to that reported for solids (Lin et al., 2007).

**Figure 7:**
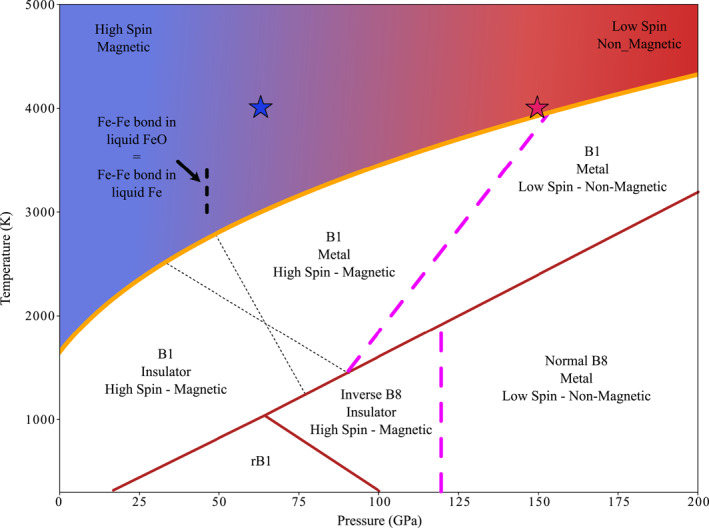
FeO phase diagram indicating the electronic and magnetic states. The different boundaries in solid FeO are from the most recent literature (Fischer, Campbell, Lord, et al., 2011; Greenberg et al., 2020; Ohta et al., 2012; Ozawa, Hirose, et al., 2011; Ozawa, Takahashi, et al., 2011). The different structural transitions are indicated in full red lines (Ozawa, Takahashi, et al., 2011). The melting curve is drawn following Figure 1 of the present study. The proposed metal to insulator isostructural transition is indicated as black thin dotted lines (Fischer, Campbell, Lord, et al., 2011; Ohta et al., 2012). The location of the end of the high spin to low spin transition is indicated by the thick purple dashed lines (Greenberg et al., 2020; Ozawa, Hirose, et al., 2011). The two stars along the 4000 K isotherm in the liquid state indicate the pressure range over which the density estimated according the thermodynamic EoS is between those expected according magnetic and non‐magnetic ab initio calculations (see Figure 6). This suggest a gradual spin state crossover occurring between ∼60 GPa (blue star) and ∼150 GPa (red star). The pressure for which the Fe‐Fe bond lengths in liquid FeO approaches that in liquid Fe is indicated by the dashed vertical bar around 50 GPa (see also Figure 5).

High‐spin to low‐spin state transition in solid FeO is expected to start at a given pressure and, depending on temperature, to span over a finite pressure range. At ambient temperature, the spin crossover pressure range span between 103 and 119 GPa, and the transition is reported to come with a 2.5% volume reduction (Ozawa, Hirose, et al., 2011). Volume reduction diminishes to ∼1% for temperature of ∼1600 K (Ozawa, Hirose, et al., 2011). Recent ab initio molecular dynamics simulations and experiments at high temperature (Greenberg et al., 2020) indicate a positive slope of the spin transition, with high spin state stable over a larger pressure range at temperatures approaching the melting (Figure 7). This opens to the possibility of metallic FeO in the high‐spin state, implying a decorrelation between spin transition and metal/insulator transition. Different combinations are possible, as the Mott transition is a transition from an antiferromagnetic insulator to a metal (which can still be antiferromagnetic) (Mott, 1990). While suggestive, this speculation appears not supported by the FeO behavior at ambient temperature, for which spin transition and metalization are reported to be concomitant (Ozawa, Hirose, et al., 2011). Further studies will then be necessary to clarify on the complex structural and electronic behavior of solid FeO at high pressure and high temperature.

In absence of experiments measuring the electrical conductivity of the liquid state, we can only indirectly assess its insulating or metallic nature, using the solid phase as guidance. The decrease in the Fe‐Fe bond length in liquid FeO with increasing pressure is characterized by two pressure ranges: between 0 and ∼40 GPa the Fe‐Fe bond length strongly decreases, followed by a more gradual decrease at higher pressure (Figures 5 and 7). This change in compressibility regime in the liquid state occurs at a pressure corresponding to that of the insulator to metal transition in solid FeO B1 structure. Indeed, depending on the considered studies (Fischer, Campbell, Lord, et al., 2011; Ohta et al., 2012), the boundary between the two conductive states intersects the melting curve at pressures between 30 and 50 GPa (Figure 1).

Up to around 60 GPa, the density according to thermodynamic EoS is in good agreement with density calculated by AIMD for magnetic/high‐spin liquid (Figures 6 and 7). Above this pressure, AIMD densities diverge from those estimated according the thermodynamic EoS, and this could be interpreted as the beginning of the transition toward a low spin state. This transition seems to expand up to ∼150 GPa, where the thermodynamic EoS crosses the non‐magnetic/low spin EoS (Figures 6 and 7). Therefore our different experimental observations, calculations and modeling argue for a liquid FeO continuously evolving from low spin to high spin in the 60–150 GPa pressure range (Figure 7), with an onset potentially triggered by the insulator/metal transition. Intriguingly, this pressure range corresponds to that over which the stability of a high spin conductive state of FeO in the B1 structure has been recently postulated (Greenberg et al., 2020) (Figure 7).

The considerations outlined above support a more complex correlation between the spin crossover and the insulator to metal transition in liquid FeO than the mere co‐occurrence, as also recently postulated for solid FeO (Greenberg et al., 2020). Specifically, the pressure‐induced transition to a metallic state strongly affects the local structure of the liquid, and could also initiate a continuous transition toward the low spin state, as in liquid FeSiO_3_ (Sun et al., 2019), completed only around 150 GPa. A full rationalization of magnetic and spin state correlation of FeO in the liquid state would however require future measurements and/or dedicated calculations of the electronic state of liquid FeO as a function of pressure and temperature.

### 4.2 Closure of the Fe‐FeO Liquid Miscibility Gap

Magnetic transitions in solid iron alloys have been observed to reflect on the corresponding liquid state. For example, the collapse of magnetic moment in solid Fe_3_S around 20 GPa (Lin et al., 2004) coincides with a structural modification toward a more compact structure in liquid Fe‐S alloys (Morard et al., 2007), and the closure of the miscibility gap in ternary Fe‐S‐Si system (Morard & Katsura, 2010; Morard et al., 2008).

**Figure 8:**
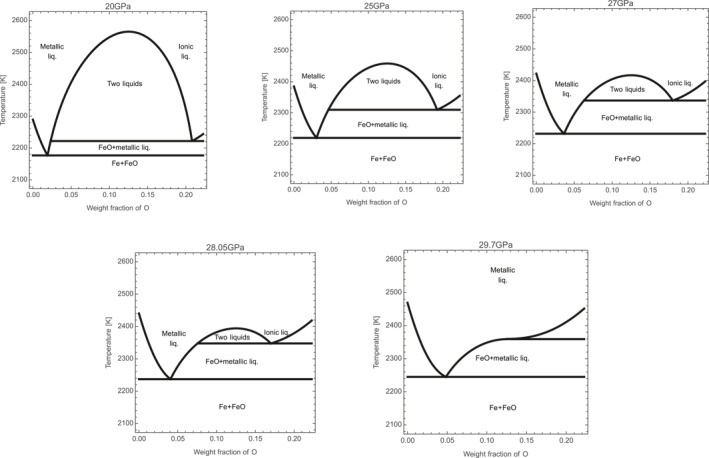
Closure of the miscibility gap in the binary Fe‐FeO phase diagram with increasing pressure. Thermodynamic calculations performed using the present data on FeO end‐member (solid and liquid EoS), the Fe end‐member EoS from Komabayashi (2014) and mixing model from Tsuno, Ohtani, and Terasaki (2007).

The results obtained in this study relate modifications at the microscopic scale with changes in the macroscopic behavior of the Fe‐FeO binary system. In this binary system, a miscibility gap is present between metallic Fe‐rich liquid and ionic FeO‐rich liquid at ambient pressure up to ∼2800 K. The miscibility gap is still relatively large at 15–20 GPa (Ringwood & Hibberson, 1990; Tsuno, Ohtani, & Terasaki, 2007) (Figure 8). Thermodynamics calculations presented here (Figure 8) based on parameters from different studies (Komabayashi, 2014; Tsuno, Ohtani, & Terasaki, 2007) and the EoS of solid and liquid FeO from this study, evidence the closure of the miscibility gap with increasing pressure. Indications of immiscibility were recently reported up to 29 GPa, and an eutectic behavior observed at 44 GPa (Oka et al., 2019). More experimental investigations of the Fe‐FeO binary system in the pressure range 25–50 GPa are therefore required to accurately determine the transition between immiscibility and eutectic behavior.

Comparison of Fe‐Fe bond lengths between liquid Fe and liquid FeO documented here (Figure 5) shows that the two liquids have similar Fe‐Fe bond lengths at about 40 GPa. This illustrates the structural compatibility between the two liquids at that pressure and is likely related to the closure of the miscibility gap in the Fe‐O binary system.

The change of structure in the liquid could play an important role in controlling various material's properties with geophysical or geochemical implications, such as the liquid‐solid partitioning of Fe during magma ocean crystallization, or favoring chemical stratification in the core of smaller planetary bodies such as Mars compared to more massive planets like the Earth.

### 4.3 Potential Benchmark for Numerical Simulations

It is interesting to discuss the current limitations in the numerical simulations based on molecular dynamics, evidenced by comparison to experimental results and thermodynamic models. These are somehow peculiar to the FeO system and all related to the treatment of the correlation effects with the GGA + *U* method. First, the value of *U* can depend on different parameters (pressure, spin state or structure) (Sun et al., 2020), implying a pressure parametrization (value chosen here is 5 eV). Calculations with *U* = 7 eV result in a pressure increase of 5 GPa at 7 g/cm^3^, a value that, however, is strongly dependent on the magnetic state, and calculations using either *U* = 5 eV or *U* = 7 eV can reach a difference of up to 20 GPa at higher densities. Another potential source of error comes from the presence of metastable states due to the several possibilities of filling the correlated electronic orbitals (3*d* for iron). Different occupation matrices can indeed have an important effect on structural properties as the equilibrium volume, see for example, a recent work on AmO_2_ (Talla Noutack et al., 2019). A solution is to try all filling configurations of the orbital via the occupation matrix control scheme (Jomard et al., 2008), as done for instance to find the ground state. Unfortunately, this solution is impossible to apply in molecular dynamics, especially for liquids where the atomic environment, and therefore the occupation matrix, of each atom is different and changes drastically during the simulation. Finally, we can also mention that the GGA + *U* is a static approximation and a more realistic methods such as dynamical mean field theory (DMFT) should be used (Leonov, 2015; Zhang et al., 2017) to describe the 3*d* electrons behavior, but the computational time of this method prevents its use for supercells and AIMD calculations.

Magnetic properties of FeO are extremely complex to simulate by ab initio calculations, as for most systems with correlated electrons. Evolution of the spin state of Fe in the liquid state is up to now poorly understood. Our experimental results illustrate that electronic properties evolves continuously between 40 GPa up to over 150 GPa, and that they are important to reproduce the expected density of liquid FeO under Earth's core‐mantle boundary conditions (Figure 6). The present experimental results could be used to potentially benchmark future ab initio calculations using more sophisticated methods to describe the 3*d* electrons behavior. More generally, magnetic effects in FeO bearing liquids, such as those related to the spin transition in liquid (Mg,Fe)O (Holmstrom & Stixrude, 2016) have to be understood, and correctly accounted for, in order to reproduce the structural properties here measured.

## 5 Conclusion

Combining in situ XRD experimental measurements, AIMD calculations and thermodynamic modeling, we discuss the properties of liquid FeO under extreme pressures, both at the atomic and macroscopic scales, with a focus of the evolution with increasing pressure, up to conditions existing at the Earth's core‐mantle boundary. Pressure‐induced changes are intimately related with electronic properties, with a possible decorrelation between the insulator‐to‐metal transition likely occurring around 40 GPa, and the spin crossover, spanning between 60 to above 150 GPa. Modifications of the local structure of liquid FeO, with Fe‐Fe bonding approaching the value in liquid Fe, explain the closure of the Fe‐FeO miscibility gap at about 40 GPa.

Experimental measurements of the melting curve and of the thermal equation of state of solid FeO in the B1 structure are used for thermodynamic modeling and the determination of the equation of state of liquid FeO.

Beside geophysical potential implications, these results are of high interest to benchmark future ab initio calculations on FeO‐bearing liquids under extreme *P*‐*T* conditions.

## Supporting information

Supporting Information S1

Supporting Information S2

## Data Availability

Analyzed data set are available as Supplementary Materials following the main article and also following the link https://doi.org/10.6084/m9.figshare.21383655.v1.

## References

[jgrb55926-bib-0001] Antonangeli, D. , Siebert, J. , Aracne, C. M. , Farber, D. L. , Bosak, A. , Hoesh, M. , et al. (2011). Spin crossover in ferropericlase at high pressure: A seismologically transparent transition? Science, 331(6013), 64–67. 10.1126/science.1198429 21212352

[jgrb55926-bib-0002] Badro, J. (2014). Spin transitions in mantle minerals. Annual Review of Earth and Planetary Sciences, 42(1), 231–248. 10.1146/annurev-earth-042711-105304

[jgrb55926-bib-0003] Badro, J. , Cote, A. S. , & Brodholt, J. P. (2014). A seismologically consistent compositional model of Earth’s core. Proceedings of the National Academy of Sciences, 111(21), 7542–7545. 10.1073/pnas.1316708111 PMC4040578 24821817

[jgrb55926-bib-0004] Boukaré, C. , Ricard, Y. , & Fiquet, G. (2015). Thermodynamics of the MgO‐FeO‐SiO_2_ system up to 140 GPa: Application to the crystallization of Earth’s magma ocean. Journal of Geophysical Research: Solid Earth, 120(9), 6085–6101. 10.1002/2015JB011929

[jgrb55926-bib-0005] Campbell, A. J. , Danielson, L. , Righter, K. , Seagle, C. T. , Wang, Y. , & Prakapenka, V. B. (2009). High pressure effects on the iron‐iron oxide and nickel‐nickel oxide oxygen fugacity buffers. Earth and Planetary Science Letters, 286(3–4), 556–564. 10.1016/j.epsl.2009.07.022

[jgrb55926-bib-0006] Crowhurst, J. C. , Brown, J. M. , Goncharov, A. F. , & Jacobsen, S. D. (2008). Elasticity of (Mg, Fe) O through the spin transition of iron in the lower mantle. Science, 319(5862), 451–453. 10.1126/science.1149606 18218893

[jgrb55926-bib-0007] Dewaele, A. , Belonoshko, A. B. , Garbarino, G. , Occelli, F. , Bouvier, P. , Hanfland, M. , & Mezouar, M. (2012). High‐pressure‐high‐temperature equation of state of KCl and KBr. Physical Review B: Condensed Matter and Materials Physics, 85(21), 214105. 10.1103/PhysRevB.85.214105

[jgrb55926-bib-0008] Dewaele, A. , Loubeyre, P. , Occelli, F. , Mezouar, M. , Dorogokupets, P. I. , & Torrent, M. (2006). Quasihydrostatic equation of state of iron above 2 Mbar. Physical Review Letters, 97(21), 29–32. 10.1103/PhysRevLett.97.215504 17155749

[jgrb55926-bib-0009] Dorogokupets, P. I. , Dymshits, A. M. , Litasov, K. D. , & Sokolova, T. S. (2017). Thermodynamics and equations of state of iron to 350 GPa and 6000 K. Scientific Reports, 7, 1–11. 10.1038/srep41863 28262683 PMC5338021

[jgrb55926-bib-0010] Eggert, J. , Weck, G. , Loubeyre, P. , & Mezouar, M. (2002). Quantitative structure factor and density measurements of high‐pressure fluids in diamond anvil cells by X‐ray diffraction: Argon and water. Physical Review B: Condensed Matter, 65(17), 1–12. 10.1103/PhysRevB.65.174105

[jgrb55926-bib-0011] Fischer, R. A. , & Campbell, A. J. (2010). High‐pressure melting of wustite. American Mineralogist, 95(10), 1473–1477. 10.2138/am.2010.3463

[jgrb55926-bib-0012] Fischer, R. A. , Campbell, A. J. , Lord, O. T. , Shofner, G. A. , Dera, P. , & Prakapenka, V. B. (2011). Phase transition and metallization of FeO at high pressures and temperatures. Geophysical Research Letters, 38(24), 2–6. 10.1029/2011GL049800

[jgrb55926-bib-0013] Fischer, R. A. , Campbell, A. J. , Shofner, G. A. , Lord, O. T. , Dera, P. , & Prakapenka, V. B. (2011). Equation of state and phase diagram of FeO. Earth and Planetary Science Letters, 304(3–4), 496–502. 10.1016/j.epsl.2011.02.025

[jgrb55926-bib-0014] Fischer, R. A. , Nakajima, Y. , Campbell, A. J. , Frost, D. J. , Harries, D. , Langenhorst, F. , et al. (2015). High pressure metal–silicate partitioning of Ni, Co, V, Cr, Si, and O. Geochimica et Cosmochimica Acta, 167, 177–194. 10.1016/j.gca.2015.06.026

[jgrb55926-bib-0015] Giampaoli, R. , Kantor, I. , Mezouar, M. , Boccato, S. , Rosa, A. D. , Torchio, R. , et al. (2018). Measurement of temperature in the laser heated diamond anvil cell: Comparison between reflective and refractive optics. High Pressure Research, 38, 250–269.

[jgrb55926-bib-0016] Gonze, X. , Amadon, B. , Antonius, G. , Arnardi, F. , Baguet, L. , Beuken, J. M. , et al. (2020). The ABINITproject: Impact, environment and recent developments. Computer Physics Communications, 248, 107042. 10.1016/j.cpc.2019.107042

[jgrb55926-bib-0017] Gramsch, S. A. , Cohen, R. E. , & Savrasov, S. Y. (2003). Structure, metal‐insulator transitions, and magnetic properties of FeO at high pressures. American Mineralogist, 88(2–3), 257–261. 10.2138/am-2003-2-301

[jgrb55926-bib-0018] Greenberg, E. , Nazarov, R. , Landa, A. , Ying, J. , Hood, R. Q. , Hen, B. , et al. (2020). Phase transitions and spin‐state of iron in FeO at the conditions of Earth’s deep interior. Arxiv.

[jgrb55926-bib-0019] Helffrich, G. , Hirose, K. , & Nomura, R. (2020). Thermodynamical modeling of liquid Fe‐Si‐Mg‐O: Molten magnesium silicate release from the core. Geophysical Research Letters, 47(21), 1–9. 10.1029/2020GL089218

[jgrb55926-bib-0020] Hirose, K. , Labrosse, S. , & Hernlund, J. (2013). Composition and state of the core. Annual Review of Earth and Planetary Sciences, 41(1), 657–691. 10.1146/annurev-earth-050212-124007

[jgrb55926-bib-0021] Hirose, K. , Morard, G. , Sinmyo, R. , Umemoto, K. , Hernlund, J. , Helffrich, G. , & Labrosse, S. (2017). SiO_2_ crystallization and compositional evolution of the Earth’s core. Nature, 543(7643), 99–102. 10.1038/nature21367 28225759

[jgrb55926-bib-0022] Holmström, E. , & Stixrude, L. (2015). Spin crossover in ferropericlase from first‐principles molecular dynamics. Physical Review Letters, 114(11), 1–5. 10.1103/PhysRevLett.114.117202 25839305

[jgrb55926-bib-0023] Holmstrom, E. , & Stixrude, L. (2016). Spin crossover in liquid (Mg, Fe) O at extreme conditions. Physical Review B: Condensed Matter and Materials Physics, 93(19), 1–7. 10.1103/PhysRevB.93.195142

[jgrb55926-bib-0024] Hume‐Rothery, W. , & Powell, H. M. (1935). On the theory of super‐lattice structures in alloys. Zeitschrift für Kristallographie ‐ Crystalline Materials, 91(1–6), 23–47. 10.1524/zkri.1935.91.1.23

[jgrb55926-bib-0025] Ita, J. , & Stixrude, L. (1992). Petrology, elasticity, and composition of the mantle transition zone. Journal of Geophysical Research, 97(B5), 6849–6866. 10.1029/92JB00068

[jgrb55926-bib-0026] Jomard, G. , Amadon, B. , Bottin, F. , & Torrent, M. (2008). Structural, thermodynamic, and electronic properties of plutonium oxides from first principles. Physical Review B: Condensed Matter and Materials Physics, 78(7), 1–11. 10.1103/PhysRevB.78.075125

[jgrb55926-bib-0027] Komabayashi, T. (2014). Thermodynamics of melting relations in the system Fe‐FeO at high pressure: Implications for oxygen in the Earth’s core. Journal of Geophysical Research: Solid Earth, 119(5), 4164–4177. 10.1002/2014JB010980.Received

[jgrb55926-bib-0028] Kuwayama, Y. , Morard, G. , Nakajima, Y. , Hirose, K. , Baron, A. Q. R. , Kawaguchi, S. I. , et al. (2020). Equation of state of liquid iron under extreme conditions. Physical Review Letters, 124(16), 165701. 10.1103/PhysRevLett.124.165701 32383924

[jgrb55926-bib-0029] Leonov, I. (2015). Metal‐insulator transition and local‐moment collapse in FeO under pressure. Physical Review B: Condensed Matter and Materials Physics, 92(8), 1–6. 10.1103/PhysRevB.92.085142

[jgrb55926-bib-0030] Leydier, M. (2010). Méthodes complémentaires pour l’étude de verres et liquides fondus sur grands instruments: Structure et dynamique. Université d'Orléans.

[jgrb55926-bib-0031] Lin, J. F. , Fei, Y. , Sturhahn, W. , Zhao, J. , Mao, H. K. , & Hemley, R. J. (2004). Magnetic transition and sound velocities of Fe_3_S at high pressure: Implications for Earth and planetary cores. Earth and Planetary Science Letters, 226(1–2), 33–40. 10.1016/j.epsl.2004.07.018

[jgrb55926-bib-0032] Lin, J. F. , Speziale, S. , Mao, Z. , & Marquardt, H. (2013). Effects of the electronic spin transitions of iron in lower mantle minerals: Implications for deep mantle geophysics and geochemistry. Reviews of Geophysics, 51(2), 244–275. 10.1002/rog.20010

[jgrb55926-bib-0033] Lin, J. F. , Vankó, G. , Jacobsen, S. D. , Iota, V. , Struzhkin, V. V. , Prakapenka, V. B. , et al. (2007). Spin transition zone in Earth’s lower mantle. Science, 317(5845), 1740–1743. 10.1017/cbo9781139167291.033 17885134

[jgrb55926-bib-0034] Marquardt, H. , Buchen, J. , Mendez, A. S. J. , Kurnosov, A. , Wendt, M. , Rothkirch, A. , et al. (2018). Elastic softening of (Mg_0.8_Fe_0.2_)O ferropericlase across the iron spin crossover measured at seismic frequencies. Geophysical Research Letters, 45(14), 6862–6868. 10.1029/2018GL077982

[jgrb55926-bib-0035] Mazin, I. , & Anisimov, V. (1997). Insulating gap in FeO: Correlations and covalency. Physical Review B: Condensed Matter and Materials Physics, 55(19), 12822–12825. 10.1103/PhysRevB.55.12822

[jgrb55926-bib-0036] McCammon, C. A. , & Liu, L. (1984). The effects of pressure and temperature on nonstoichiometric wustite, FexO: The iron‐rich phase boundary. Physics and Chemistry of Minerals, 10(3), 106–113. 10.1007/bf00309644

[jgrb55926-bib-0037] Mezouar, M. , Crichton, W. A. , Bauchau, S. , Thurel, F. , Witsch, H. , Torrecillas, F. , et al. (2005). Development of a new state‐of‐the‐art beamline optimized for monochromatic single crystal and powder X‐ray diffraction under extreme conditions at the ESRF. Journal of Synchrotron Radiation, 12(5), 659–664. 10.1107/s0909049505023216 16120991

[jgrb55926-bib-0038] Mezouar, M. , Faure, P. , Crichton, W. , Rambert, N. , Sitaud, B. , Bauchau, S. , & Blattmann, G. (2002). Multichannel collimator for structural investigation of liquids and amorphous materials at high pressures and temperatures. Review of Scientific Instruments, 73(10), 3570–3574. 10.1063/1.1505104

[jgrb55926-bib-0039] Mezouar, M. , Giampaoli, R. , Garbarino, G. , Kantor, I. , Dewaele, A. , Weck, G. , et al. (2017). Methodology for in situ synchrotron X‐ray studies in the laser‐heated diamond anvil cell. High Pressure Research, 37(2), 170–180. 10.1080/08957959.2017.1306626

[jgrb55926-bib-0040] Misawa, M. , Ohmura, S. , & Shimojo, F. (2014). Structural properties of Fe_2_O_3_ at high temperatures. Journal of the Physical Society of Japan, 83(10), 105002. 10.7566/jpsj.83.105002

[jgrb55926-bib-0041] Morard, G. , Andrault, D. , Antonangeli, D. , Nakajima, Y. , Auzende, A. L. , Boulard, E. , et al. (2017). Fe–FeO and Fe–Fe_3_C melting relations at Earth’s core–mantle boundary conditions: Implications for a volatile‐rich or oxygen‐rich core. Earth and Planetary Science Letters, 473, 94–103. 10.1016/j.epsl.2017.05.024

[jgrb55926-bib-0042] Morard, G. , Bouchet, J. , Rivoldini, A. , Antonangeli, D. , Roberge, M. , Boulard, E. , et al. (2018). Liquid properties in the Fe‐FeS system under moderate pressure: Tool box to model small planetary cores. American Mineralogist, 103, 1770–1779. 10.2138/am-2018-6405

[jgrb55926-bib-0043] Morard, G. , Garbarino, G. , Antonangeli, D. , Andrault, D. , Guignot, N. , Siebert, J. , et al. (2013). Density measurements and structural properties of liquid and amorphous metals under high pressure. High Pressure Research, 00, 1–13. 10.1080/08957959.2013.860137

[jgrb55926-bib-0044] Morard, G. , & Katsura, T. (2010). Pressure‐temperature cartography of Fe‐S‐Si immiscible system. Geochimica et Cosmochimica Acta, 74(12), 3659–3667. 10.1016/j.gca.2010.03.025

[jgrb55926-bib-0045] Morard, G. , Mezouar, M. , Bauchau, S. , Alvarez‐Murga, M. , Hodeau, J.‐L. , & Garbarino, G. (2011). High efficiency multichannel collimator for structural studies of liquids and low‐Z materials at high pressures and temperatures. Review of Scientific Instruments, 82(2), 023904. 10.1063/1.3551988 21361608

[jgrb55926-bib-0046] Morard, G. , Nakajima, Y. , Andrault, D. , Antonangeli, D. , Auzende, A. L. , Boulard, E. , et al. (2017). Structure and density of Fe‐C liquid alloys under high pressure. Journal of Geophysical Research: Solid Earth, 122(10), 1–11. 10.1002/2017JB014779

[jgrb55926-bib-0047] Morard, G. , Sanloup, C. , Fiquet, G. , Mezouar, M. , Rey, N. , Poloni, R. , & Beck, P. (2007). Structure of eutectic Fe‐FeS melts to pressures up to 17 GPa: Implications for planetary cores. Earth and Planetary Science Letters, 263(1–2), 128–139. 10.1016/j.epsl.2007.09.009

[jgrb55926-bib-0048] Morard, G. , Sanloup, C. , Guillot, B. , Fiquet, G. , Mezouar, M. , Perrillat, J. P. , et al. (2008). In situ structural investigation of Fe‐S‐Si immiscible liquid system and evolution of Fe‐S bond properties with pressure. Journal of Geophysical Research, 113(B10), B10205. 10.1029/2008JB005663

[jgrb55926-bib-0049] Mott, N. F. (1990). Metal‐insulator transitions (2nd ed.).

[jgrb55926-bib-0050] Ohta, K. , Cohen, R. E. , Hirose, K. , Haule, K. , Shimizu, K. , & Ohishi, Y. (2012). Experimental and theoretical evidence for pressure‐induced metallization in FeO with rocksalt‐type structure. Physical Review Letters, 108(2), 1–5. 10.1103/PhysRevLett.108.026403 22324701

[jgrb55926-bib-0051] Oka, K. , Hirose, K. , Tagawa, S. , Kidokoro, Y. , Nakajima, Y. , Kuwayama, Y. , et al. (2019). Melting in the Fe‐FeO system to 204 GPa: Implications for oxygen in Earth’s core. American Mineralogist, 104(11), 1603–1607. 10.2138/am-2019-7081

[jgrb55926-bib-0052] Ozawa, H. , Hirose, K. , Ohta, K. , Ishii, H. , Hiraoka, N. , Ohishi, Y. , & Seto, Y. (2011). Spin crossover, structural change, and metallization in NiAs‐type FeO at high pressure. Physical Review B: Condensed Matter and Materials Physics, 84(13), 1–6. 10.1103/PhysRevB.84.134417

[jgrb55926-bib-0053] Ozawa, H. , Takahashi, F. , Hirose, K. , Ohishi, Y. , & Hirao, N. (2011). Phase transition of FeO and stratification in Earth’s outer core. Science, 334(6057), 792–794. 10.1126/science.1208265 22076374

[jgrb55926-bib-0054] Poirier, J. P. (1994). Light elements in the Earth’s outer core: A critical review. Physics of the Earth and Planetary Interiors, 85(3–4), 319–337. 10.1016/0031-9201(94)90120-1

[jgrb55926-bib-0055] Poirier, J. P. (2000). Introduction to the physics of the Earth’s interior.

[jgrb55926-bib-0056] Prescher, C. , Prakapenka, V. B. , Stefanski, J. , Jahn, S. , Skinner, L. B. , & Wang, Y. (2017). Beyond sixfold coordinated Si in SiO_2_ glass at ultrahigh pressures. Proceedings of the National Academy of Sciences, 114(38), 10041–10046. 10.1073/pnas.1708882114 PMC5617297 28874582

[jgrb55926-bib-0057] Rai, N. , & Van Westrenen, W. (2014). Lunar core formation: New constraints from metal‐silicate partitioning of siderophile elements. Earth and Planetary Science Letters, 388, 343–352. 10.1016/j.epsl.2013.12.001

[jgrb55926-bib-0058] Righter, K. , & Chabot, N. L. (2011). Moderately and slightly siderophile element constraints on the depth and extent of melting in early Mars. Meteoritics & Planetary Science, 46(2), 157–176. 10.1111/j.1945-5100.2010.01140.x

[jgrb55926-bib-0059] Ringwood, A. E. , & Hibberson, W. (1990). The system Fe‐FeO revisited. Physics and Chemistry of Minerals, 17(4), 313–319. 10.1007/bf00200126

[jgrb55926-bib-0060] Sanloup, C. , & Fei, Y. (2004). Closure of the Fe‐S‐Si liquid miscibility gap at high pressure. Physics of the Earth and Planetary Interiors, 147(1), 57–65. 10.1016/j.pepi.2004.06.008

[jgrb55926-bib-0061] Shen, G. , Prakapenka, V. B. , Eng, P. J. , Rivers, M. L. , & Sutton, S. R. (2006). Facilities for high‐pressure research with the diamond anvil cell at GSECARS SXD at Mbar pressures. Journal of Synchrotron Radiation, 12(5), 642–649. 10.1107/S0909049505022442 16120989

[jgrb55926-bib-0062] Siebert, J. , Badro, J. , Antonangeli, D. , & Ryerson, F. J. (2013). Terrestrial accretion under oxidizing conditions. Science, 339(6124), 1194–1197. 10.1126/science.1227923 23306436

[jgrb55926-bib-0063] Simon, F. , & Glatzel, G. (1929). Bernerkungen zur Schmelzdruckkurve. Zeitschrift fuer Anorganische und Allgemeine Chemie, 178(1), 309–316. 10.1002/zaac.19291780123

[jgrb55926-bib-0064] Skinner, L. B. , Huang, C. , Schlesinger, D. , Pettersson, L. G. M. , Nilsson, A. , & Benmore, C. J. (2013). Benchmark oxygen‐oxygen pair‐distribution function of ambient water from x‐ray diffraction measurements with a wide Q‐range. Journal of Chemical Physics, 138(7), 074506. 10.1063/1.4790861 23445023

[jgrb55926-bib-0065] Stähler, S. C. , Khan, A. , Bruce Banerdt, W. , Lognonné, P. , Giardini, D. , Ceylan, S. , et al. (2021). Seismic detection of the Martian core. Science, 373(6553), 443–448. 10.1126/science.abi7730 34437118

[jgrb55926-bib-0066] Stixrude, L. , & Lithgow‐Bertelloni, C. (2007). Influence of phase transformations on lateral heterogeneity and dynamics in Earth’s mantle. Earth and Planetary Science Letters, 263(1–2), 45–55. 10.1016/j.epsl.2007.08.027

[jgrb55926-bib-0067] Sun, Y. , Cococcioni, M. , & Wentzcovitch, R. M. (2020). LDA + *U* _ *sc* _ calculations of phase relations in FeO. Physical Review Materials, 4(6), 63605. 10.1103/PhysRevMaterials.4.063605

[jgrb55926-bib-0068] Sun, Y. , Zhou, H. , Yin, K. , & Lu, X. (2019). First‐principles study of thermodynamics and spin transition in FeSiO_3_ liquid at high pressure. Geophysical Research Letters, 46(7), 3706–3716. 10.1029/2018GL081421

[jgrb55926-bib-0069] Talla Noutack, M. S. , Geneste, G. , Jomard, G. , & Freyss, M. (2019). First‐principles investigation of the bulk properties of americium dioxide and sesquioxides. Physical Review Materials, 3, 1–13. 10.1103/PhysRevMaterials.3.035001

[jgrb55926-bib-0070] Trønnes, R. G. , Baron, M. A. , Eigenmann, K. R. , Guren, M. G. , Heyn, B. H. , Løken, A. , & Mohn, C. E. (2019). Core formation, mantle differentiation and core‐mantle interaction within Earth and the terrestrial planets. Tectonophysics, 760, 1–34. 10.1016/j.tecto.2018.10.021

[jgrb55926-bib-0071] Tsuno, K. , Frost, D. J. , & Rubie, D. C. (2011). The effects of nickel and sulphur on the core‐mantle partitioning of oxygen in Earth and Mars. Physics of the Earth and Planetary Interiors, 185(1–2), 1–12. 10.1016/j.pepi.2010.11.009

[jgrb55926-bib-0072] Tsuno, K. , Ohtani, E. , & Terasaki, H. (2007). Immiscible two‐liquid regions in the Fe‐O‐S system at high pressure: Implications for planetary cores. Physics of the Earth and Planetary Interiors, 160(1), 75–85. 10.1016/j.pepi.2006.09.004

[jgrb55926-bib-0073] Tsuno, K. , Terasaki, H. , Ohtani, E. , Suzuki, A. , Asahara, Y. , Nishida, K. , et al. (2007). In situ observation and determination of liquid immiscibility in the Fe‐O‐S melt at 3 GPa using a synchrotron X‐ray radiographic technique. Geophysical Research Letters, 34(17), L17303. 10.1029/2007GL030750

[jgrb55926-bib-0074] Waseda, Y. (1980). The structure of non crystalline materials.

[jgrb55926-bib-0075] Waseda, Y. , & Suzuki, K. (1970). Atomic distribution and magnetic moment in liquid iron by neutron diffraction. Physica Status Solidi, 39(2), 669–678. 10.1002/pssb.19700390235

[jgrb55926-bib-0076] Weck, G. , Garbarino, G. , Ninet, S. , Spaulding, D. , Datchi, F. , Loubeyre, P. , & Mezouar, M. (2013). Use of a multichannel collimator for structural investigation of low‐Z dense liquids in a diamond anvil cell: Validation on fluid H_2_ up to 5 GPa. Review of Scientific Instruments, 84(6), 063901. 10.1063/1.4807753 23822351

[jgrb55926-bib-0077] Wojdyr, M. (2010). Fityk: A general‐purpose peak fitting program. Journal of Applied Crystallography, 43(5), 1126–1128. 10.1107/S0021889810030499

[jgrb55926-bib-0078] Xin, J. , Gan, L. E. I. , Wang, N. A. N. , & Chen, M. I. N. (2019). Accurate density calculation for molten slags in SiO_2_‐Al_2_O_3_‐CaO‐MgO‐‘FeO’‐‘Fe_2_O_3_’ systems. Metallurgical and Materials Transactions B, 50(6), 2828–2842. 10.1007/s11663-019-01674-1

[jgrb55926-bib-0079] Yoshizaki, T. , & McDonough, W. F. (2020). The composition of Mars. Geochimica et Cosmochimica Acta, 273, 137–162. 10.1016/j.gca.2020.01.011

[jgrb55926-bib-0080] Zhang, P. , Cohen, R. E. , & Haule, K. (2017). Magnetic phase diagram of FeO at high pressure. Journal of Physics: Conference Series, 827, 012006. 10.1088/1742-6596/827/1/012006

