# Supplementary material for: Structural and Electronic Transitions in Liquid FeO Under High Pressure
Source: J Geophys Res Solid Earth. 2022 Nov 5;127(11):e2022JB025117. doi: 10.1029/2022JB025117 (PMC9788056; doi:10.1029/2022JB025117)
Supplement: Supplementary file 1 — Supporting Information S1 [file JGRB-127-e2022JB025117-s002.docx]

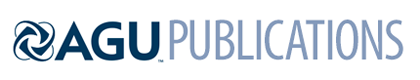


*[Journal of Geophysical Research – Solid Earth]*

Supporting Information for

**Structural and electronic transitions in liquid FeO under high pressure**

**G. Morard^a,b^, D. Antonangeli^b^, J. Bouchet^c,d^, A. Rivoldini^e^, S. Boccato^b^, F. Miozzi^b,1^, E. Boulard^b^, H. Bureau^b^, M. Mezouar^f^, C. Prescher^g^, S. Chariton^h^, E. Greenberg^h,2^**

*^a^Université Grenoble Alpes, Université Savoie Mont Blanc, CNRS, IRD, IFSTTAR, ISTerre, 38000 Grenoble, France*

*^b^Sorbonne Université, Muséum National d'Histoire Naturelle, UMR CNRS 7590, Institut de Minéralogie, de Physique des Matériaux et de Cosmochimie, IMPMC, 75005 Paris, France*

*^c^CEA, DAM, DIF, Bruyères Le Chatel, France*

*^d^CEA, DES, IRESNE, DEC, Cadarache, F-13018 St Paul Les Durance, France*

*^e^Royal Observatory of Belgium, Avenue Circulaire 3, B-1180 Brussels, Belgium*

*^f^ESRF, Grenoble, France*

*^g^DESY, Hamburg, Germany*

*^h^GSECARS, The University of Chicago, 60637, Chicago, IL, USA*

**Corresponding author. Email address: guillaume.morard@univ-grenoble-alpes.fr*

*^1^Currently at: Earth and Planets Laboratory, Carnegie Institution for Science, Washington, DC, USA*

*^2^Currently at: Applied Physics Division, Soreq NRC, Yavne, 81800, Israel*

**Contents of this file**

Figures S1 to S3

**Additional Supporting Information (Files uploaded separately)**

Tables S1 to S5

***Figure S1 : FeO B1 volume dataset with the thermal EoS.*** *This dataset combines the volumes derived from in situ XRD measurements performed here as well as from Fischer et al, 2011a. Three isotherms at 1500 K, 2000 K and 2500 K are indicated.*

***Figure S2 : Example of fit of experimental g(r) using Fityk (Wojdyr 2010).*** *The present dataset is run ESRF_S1_FeO5 #30 (35 GPa and 3000 K; Table 1). Two gaussians and one sigmoid were used to fit the g(r), over a range up to 0.4 nm.*

**

*Figure S3: O-O partial g(r) extracted from AIMD calculations. This dataset shows no evidence of O-O dimer formation.*
